# Structural basis for the subtype-selectivity of K_Ca_2.2 channel activators

**DOI:** 10.21203/rs.3.rs-6568445/v1

**Published:** 2025-05-16

**Authors:** Miao Zhang, Young-Woo Nam, Alena Ramanishka, Yang Xu, Rose Marie Yasuda, Dohyun Im, Meng Cui, George Chandy, Heike Wulff

**Affiliations:** Chapman University; Chapman University; Chapman University; SLAC National Accelerator Laboratory,; Oregon Health & Science University; Kyoto University; Northeastern University; Nanyang Technological University; University of California, Davis

## Abstract

Small-conductance (K_Ca_2.2) and intermediate-conductance (K_Ca_3.1) Ca^2+^-activated K^+^ channels are gated by a Ca^2+^-calmodulin dependent mechanism. NS309 potentiates the activity of both K_Ca_2.2 and K_Ca_3.1, while rimtuzalcap selectively activates K_Ca_2.2. Rimtuzalcap has been used in clinical trials for the treatment of spinocerebellar ataxia and essential tremor. We report cryo-electron microscopy structures of K_Ca_2.2 channels bound with NS309 and rimtuzalcap, in addition to K_Ca_3.1 channels with NS309. The different conformations of calmodulin and the cytoplasmic HC helices in the two channels underlie the subtype-selectivity of rimtuzalcap for K_Ca_2.2. Calmodulin’s N-lobes in the K_Ca_2.2 structure are far apart and undergo conformational changes to accommodate either NS309 or rimtuzalcap. Calmodulin’s Nlobes in the K_Ca_3.1 structure are closer to each other and are constrained by the HC helices of K_Ca_3.1, which allows binding of NS309 but not of the bulkier rimtuzalcap. These structures provide a framework for structure-based drug design targeting K_Ca_2.2 channels.

## Introduction

Pharmacological activation of K^+^ channels can dampen electrical signaling and may have therapeutic value for diverse diseases. Pharmacological agents that activate voltage-gated (K_V_)^1–6^, inward-rectifier (K_IR_)^7^, two-pore domain (K_2P_)^8^, and large conductance (K_Ca_1.1)^9^ K^+^ channels widen the inner gate at the intracellular entrance to the channel pore, modulate the voltage-sensing domains, or regulate gating at the selectivity filter. The mechanism underlying the action of pharmacological activators of small-conductance Ca^2+^-activated K^+^ (K_Ca_2.1-K_Ca_2.3, also called SK1-SK3) channels, an important sub-family of K^+^ channels with distinctive biophysical and pharmacological properties, has not been defined.

K_Ca_2.x channels and the related K_Ca_3.1 channel are encoded by the *KCNN1–4* gene family. K_Ca_2.x channels are critical modulators of neuronal and cardiac excitability, while KCa3.1 sustains Ca^2+^ signaling through K + efflux-driven hyperpolarization in peripheral tissues, including erythrocytes, immune cells and vascular endothelium.^10^ The shared Ca^2+^-calmodulin (CaM) dependent gating mechanism of these channels has been demonstrated by high resolution structure-determination using cryogenic electron microscopy (cryo-EM).^11,12^ In the Ca^2+^-free state, CaM’s N-lobe is flexible and invisible in the cryo-EM structure, while CaM’s C-lobe interacts with the channel’s cytoplasmic HA/HB helices (previously called CaM binding domain).^12^ During Ca^2+^-dependent activation, the calcified CaM N-lobe swings up and binds to the channel’s S4-S5 linker (primarily the S_45_A helix), causing the inner gate to open, allowing K^+^ to flow through the channel pore.^11,12^

Significant effort spanning decades has identified pharmacological activators of K_Ca_2.x and K_Ca_3.1 channels. These include compounds such as 1-EBIO, CyPPA, NS309, and GW-542573X.^10^ Earlier crystallographic studies suggested that these activators bound to the interface between CaM’s N-lobe and the channel’s cytoplasmic HA/HB helices.^13–15^ However, these studies were performed with truncated cytoplasmic HA/HB helices in complex with CaM and did not include the remainder of the channel. Their mechanism of activation therefore needs to be redefined within the context of the full-length structures. Here, we combined cryo-EM, site-directed mutagenesis, and electrophysiology to define the mechanism of action of rimtuzalcap, a CyPPA derivative that activates selectively K_Ca_2.2/K_Ca_2.3 channels, and NS309, a non-selective activator of both K_Ca_2.x and K_Ca_3.1 channels. Our studies show that both activators widen the inner gate of the K_Ca_2.2 channel, while differences in their interactions with the Ca^2+^-CaM-dependent gating machinery underlie rimtuzalcap’s selectivity for K_Ca_2.2/K_Ca_2.3 channels.

## Results

### Differential interactions of CaM with the HC helices of K_Ca_2.2 and K_Ca_3.1

**Figure 1:**
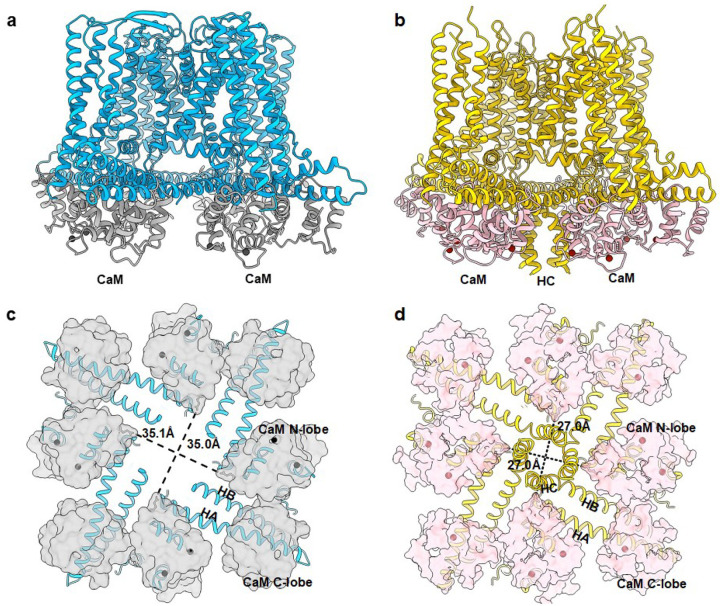
Different conformations of CaM in apo_K_Ca_2.2 and apo_K_Ca_3.1_II. **a** Side view of apo_K_Ca_2.2 (K_Ca_2.2: blue/CaM: gray) shows channel-CaM interactions. The cytoplasmic HC helices are invisible. **b** Side view of apo_K_Ca_3.1_II (K_Ca_3.1: yellow/CaM: pink, PDB 6cno) shows channel-CaM interactions. The C-terminal HC helices are visible. **c** Intracellular view of apo_K_Ca_2.2 (K_Ca_2.2: blue cartoon/CaM: grey surface). The CaM N-lobes are positioned far apart, and the HC helices are not visible probably due to flexibility. **d** Intracellular view of apo_K_Ca_3.1_II (K_Ca_3.1: yellow cartoon/CaM: pink surface). The CaM N-lobes are positioned close to each other, which stabilize the HC helices in the center.

We compared our recently determined cryo-EM structure of Ca^2+^-bound apo_K_Ca_2.2/CaM (resolution = 3.18 Å; referred to henceforth as apo_K_Ca_2.2; Fig. 1a)^11^ with the previously reported cryo-EM structure of Ca^2+^-bound apo_K_Ca_3.1/CaM activation state II^12^ (Protein Data Bank [PDB]: 6cno; referred to henceforth as apo_K_Ca_3.1_II; Fig. 1b). The cytoplasmic HC helices are invisible in the apo_K_Ca_2.2 structure possibly due to flexibility (Fig. 1a). In contrast, the C-terminal HC helices are well resolved and are seen between CaM molecules in the apo_K_Ca_3.1_II structure (Fig. 1b).

The distinct conformations of the HC helices in the two channels may be attributable to different conformations of CaM in the two structures. In apo_K_Ca_2.2, CaM’s N-lobes are too far apart (Asn42 in opposite CaM molecules are ~35.0 Å apart) to stabilize the HC helices, which may contribute to their flexibility (Fig. 1c). In the apo_K_Ca_3.1_II structure, in contrast, CaM’s N-lobes are sufficiently close (Asn42 in opposite CaM molecules are ~27.0 Å apart) to stabilize the HC helices, causing them to be visible (Fig. 1d). As will be seen below, these differences between apo_K_Ca_2.2 and apo_K_Ca_3.1_II contribute to the selectivity of rimtuzalcap for K_Ca_2.2 over K_Ca_3.1.

### The non-selective NS309 interacts similarly with K_Ca_3.1/CaM and K_Ca_2.2/CaM

**Figure 2:**
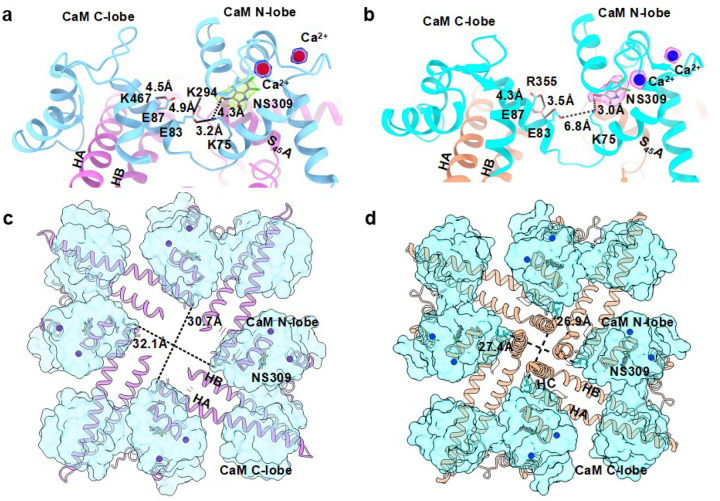
Conformation of CaM in activator-bound NS309_K_Ca_2.2 and NS309_K_Ca_3.1 structures. **a** Cryo-EM densities for Ca^2+^ and NS309 bound to one CaM molecule in NS309_K_Ca_2.2 are shown as blue and green mesh contoured at s = 6, respectively. CaM_Lys75 interacts with both CaM_Glu83 and NS309. **b** Cryo-EM densities for Ca^2+^ and NS309 bound to one CaM molecule in NS309_K_Ca_3.1 are shown as magenta mesh contoured at s = 6. CaM_Lys75 interacts with NS309 primarily. **c** Intracellular view of NS309_K_Ca_2.2 (K_Ca_2.2: purple cartoon/CaM: light blue surface). The CaM N-lobes are positioned far apart, and the HC helices are not visible probably due to flexibility. **d** Intracellular view of NS309_K_Ca_3.1 (K_Ca_3.1: salmon cartoon/CaM: cyan surface). The CaM N-lobes are positioned close to each other, which stabilize the HC helices in the center.

NS309 activates both K_Ca_3.1 (EC_50_: ~74 nM) and K_Ca_2.2 (EC_50_: ~1.7 mM) channels.^16,17^ We determined structures of NS309 bound to K_Ca_2.2/CaM and to K_Ca_3.1/CaM. The cryo-EM map of the Ca^2+^- and NS309-bound K_Ca_2.2/CaM complex (henceforth referred to as NS309_K_Ca_2.2) was refined to a resolution of 2.71 Å (SupplementaryFig. 1,2,SupplementaryTable 1). The cryo-EM map of the Ca^2+^- and NS309-bound K_Ca_3.1/CaM complex (henceforth referred to as NS309_K_Ca_3.1) was refined to a resolution of 3.59 Å (SupplementaryFig. 3,4,SupplementaryTable 1). Cryo-EM densities for NS309 in each of the four CaM’s N-lobes are clearly visible in both NS309_K_Ca_2.2 (Supplementary Fig. 5a,b) and NS309_K_Ca_3.1 (Supplementary Fig. 6a,b). We successfully built models for NS309 into the cryo-EM densities in both structures at the interface between CaM’s N-lobe and the channels’ S_45_A helix (Fig. 2a,b). Since CaM’s N-lobe forms a significant portion of NS309’s binding pocket, we compared the conformations of CaM in the apo versus activator-bound structures. CaM molecules aligned well in the apo_K_Ca_2.2 and NS309_K_Ca_2.2 comparison (rmsd = 1.4 Å, Supplementary Fig. 7a,b), and even better in the apo_K_Ca_3.1_II and NS309_K_Ca_3.1 comparison (rmsd = 1.0 Å, Supplementary Fig. 7c,d). Binding of NS309 to the K_Ca_2.2/CaM complex shortened the distance between CaM’s N-lobes from ~35.0 Å in apo_K_Ca_2.2 (Fig. 1c) to ~31.0 Å in NS309_K_Ca_2.2 (Fig. 2c). Binding of NS309 to the K_Ca_3.1/CaM complex did not affect the distance between CaM’s N-lobes (Fig. 2d) compared to the apo_K_Ca_3.1_II structure (Fig. 1d). The HC helices are visible in both apo_K_Ca_3.1_II (Fig. 1d) and NS309_K_Ca_3.1 (Fig. 2d), while they remain invisible in NS309_K_Ca_2.2 (Fig. 2c). Based on these results, we conclude that the binding pocket for NS309 preexists in both K_Ca_2.2 and K_Ca_3.1 channels.

**Figure 3:**
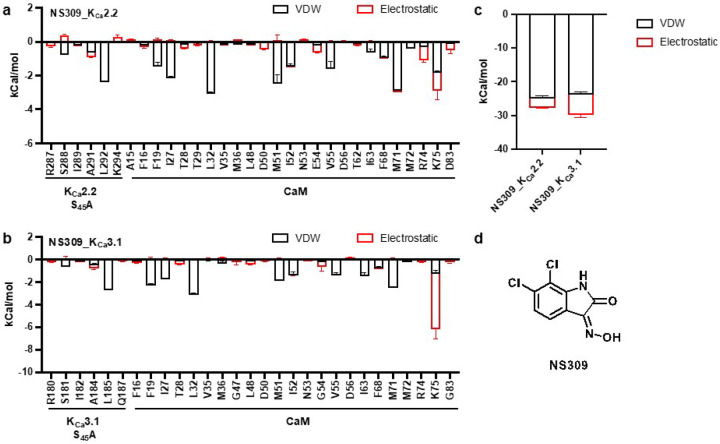
Calculated binding energy between NS309 and the binding pockets in NS309_K_Ca_2.2 and NS309_K_Ca_3.1. a Binding energy between NS309 and amino acid residues in CaM and the S_45_A helix of K_Ca_2.2. b Binding energy between NS309 and amino acid residues in CaM and the S_45_A helix of K_Ca_3.1. The total binding energy between NS309 and the four subunits of the activator-bound structure include van der Waals forces (VDW, black) and electrostatic interactions (Electrostatic, red). c Total binding energy of NS309 to binding pockets in NS309_K_Ca_2.2 and NS309_K_Ca_3.1, including van der Waals forces (VDW, black) and electrostatic interactions (Electrostatic, red). d Chemical structure of NS309.

We calculated the binding energy (van der Waals forces plus electrostatic interactions) between the four bound NS309 molecules and amino acid residues in the four subunits of the activator-bound structures of K_Ca_2.2 and K_Ca_3.1 using the Discovery Studio program. In the NS309_K_Ca_2.2 structure, NS309 sits at the interface between CaM’s N-lobe and K_Ca_2.2’s S_45_A helix, with Ser288 and Leu292 in K_Ca_2.2’s S_45_A helix interacting with NS309, and a tetrad of hydrophobic residues (Phe19, Leu32, Met51, and Met71; FLMM_N_^18^) in CaM’s N-lobe cradling the NS309 molecule (Fig. 3a). In the NS309_K_Ca_3.1 structure, NS309 fits perfectly into a hydrophobic pocket between CaM’s N-lobe and K_Ca_3.1’s S_45_A helix, with Ser181 and Leu185 in K_Ca_3.1’s S_45_A helix interacting with NS309, and the same tetrad of hydrophobic residues (Phe19, Leu32, Met51, and Met71; FLMM_N_^18^) in CaM’s N-lobe cradling the NS309 molecule (Fig. 3b). These results are consistent with earlier reports that mutations of Ser181 and Leu185 in K_Ca_3.1 and mutations of Ser288 and Leu292 in K_Ca_2.2 significantly reduced sensitivity to NS309.^17^

The total binding energies were −27.6 ± 1.0 kCal/mol and −29.7 ± 1.1 kCal/mol for NS309_K_Ca_2.2 and NS309_K_Ca_3.1, respectively (P=0.03, n=4, unpaired two-tailed Student’s *t*-test, Fig. 3c). The van der Waals forces involved in NS309’s interactions with the two channels are comparable (NS309_K_Ca_2.2: −24.9 ± 0.8 kCal/mol; NS309_K_Ca_3.1: −23.7 ± 0.7 kCal/mol; P=0.06, n=4, unpaired two-tailed Student’s *t*-test). NS309’s stronger electrostatic interactions with K_Ca_3.1 (−6.0 ± 0.7 kCal/mol) versus K_Ca_2.2 (−2.7 ± 0.3 kCal/mol, P=0.0003, n=4, unpaired two-tailed Student’s *t*-test, Fig. 3c) account for NS309’s stronger total binding energy to K_Ca_3.1 than K_Ca_2.2. In both structures, CaM_Lys75 is the largest contributor to the electrostatic interactions with NS309 (Fig. 3a,b). Electrostatic interactions between CaM_Lys75 and NS309 are stronger in NS309_K_Ca_3.1 (−4.9 ± 0.8 kCal/mol) than in NS309_K_Ca_2.2 (−1.1 ± 0.5 kCal/mol, P=0.0002, n=4, unpaired two-tailed Student’s *t*-test). NS309’s (Fig. 3d) higher binding energy to K_Ca_3.1 than K_Ca_2.2 is consistent with NS309’s ~20-fold higher potency in activating K_Ca_3.1 than K_Ca_2.2.^16,17^

In NS309_K_Ca_2.2, the compact conformation of CaM is stabilized by salt bridges between CaM’s C-lobe (Glu83 and Glu 87 in helix V) and the K_Ca_2.2 channel’s S_45_A helix (Lys294) and HB helix (Lys467) (Fig. 2a). CaM_Lys75 forms a salt bridge (~3.2 Å) with CaM_Glu83, which may weaken its hydrogen bond with NS309 (~4.3 Å, Fig. 2a). In NS309_K_Ca_3.1, CaM’s more extended conformation is stabilized by salt bridges between CaM’s C-lobe (Glu83 and Glu87 in helix V) and K_Ca_3.1’s HB helix (Arg355) (Fig. 2b). CaM_Lys75 is ~6.8 Å away from CaM_Glu83, while it is much closer to NS309 (~3.0 Å, Fig. 2b). In summary, both K_Ca_2.2 and K_Ca_3.1 can accommodate NS309 into pre-existing binding pockets at the interface between CaM’s N-lobe and the channels’ S_45_A helix, which may explain the non-selective activation of K_Ca_2.x and K_Ca_3.1 channels by NS309.^16,17^

### Structure of K_Ca_2.2/CaM bound to the subtype-selective activator rimtuzalcap

CyPPA, a positive allosteric modulator, potentiates the activity of K_Ca_2.2 and K_Ca_2.3, but is inactive on K_Ca_3.1.^19^ Rimtuzalcap is a CyPPA derivative that potentiates K_Ca_2.2 at low micromolar concentrations (EC_50_: ~5.1 mM) and is inactive on K_Ca_3.1. Rimtuzalcap was evaluated in a Phase-2 clinical trial as a treatment for essential tremor (ClinicalTrials.gov: NCT03688685), and a phase-2 clinical trial evaluating rimtuzalcap in patients with spinocerebellar ataxia was initiated (ClinicalTrials.gov: NCT03688685) and then withdrawn when Cadent Therapeutics was acquired by Novartis.

**Figure 4:**
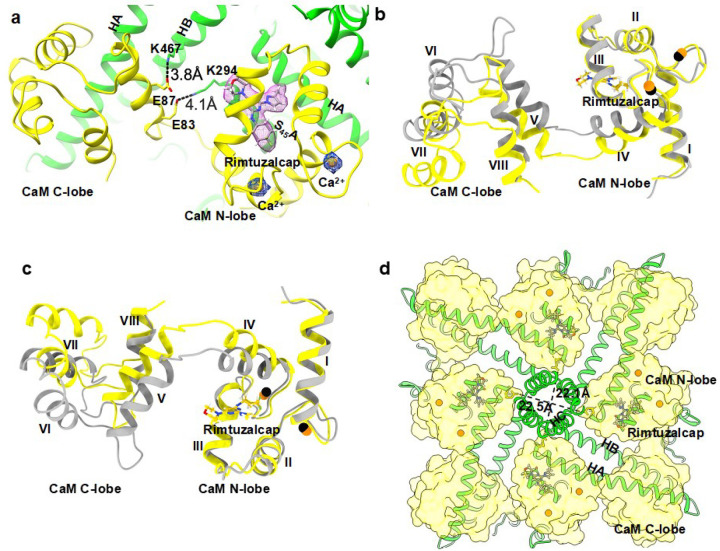
Conformation of CaM in the activator-bound rimtuzalcap_K_ca_2.2_I structure. **a** Cryo-EM densities for Ca^2+^ and rimtuzalcap bound to one CaM molecule are shown as blue and magenta mesh, respectively. The densities are contoured at s = 6. Salt bridges between helix V of CaM (Glu83 and Glu87, yellow) and the S_45_A (Lys294) and HB (Lys467) helices of K_Ca_2.2 (green) are present in the rimtuzalcap_K_Ca_2.2_I structure. **b** One Ca^2+^-bound CaM molecule of rimtuzalcap_K_Ca_2.2_I (CaM: yellow/Ca^2+^: orange) superimposed on a Ca^2+^-bound CaM molecule of apo_K_Ca_2.2 (CaM: gray/Ca^2+^: black) with the hydrophobic surfaces at the N- and C-lobes facing away. **c** One Ca^2+^-bound CaM molecule of rimtuzalcap_K_Ca_2.2_I superimposed on a Ca^2+^-bound CaM molecule of apo_K_Ca_2.2 with the hydrophobic surfaces at the N- and C-lobes facing the viewer. **d** Intracellular view of rimtuzalcap_K_Ca_2.2_I (K_Ca_2.2: green cartoon/CaM: yellow surface). Rimtuzalcap shortens the distances between the CaM N-lobes, which stabilize and make the HC helices visible.

We refined the cryo-EM map of the Ca^2+^- and rimtuzalcap-bound K_Ca_2.2/CaM (henceforth referred to as rimtuzalcap_K_Ca_2.2_I) to a resolution of 3.13 Å (SupplementaryFig. 8, 9, Supplementary Table 1). Cryo-EM densities for rimtuzalcap in each of the four CaM’s N-lobes are clearly visible in rimtuzalcap_K_Ca_2.2_I (SupplementaryFig. 10a,b). We successfully built a model for rimtuzalcap into its cryo-EM density. Rimtuzalcap sits at the interface between CaM’s N-lobe and K_Ca_2.2’s S_45_A/HA helices in each subunit of the tetrameric channel (Fig. 4a). Similar to the NS309_K_Ca_2.2 structure (Fig. 2a), the rimtuzalcap_K_Ca_2.2_I structure shows that the salt bridges between CaM helix V (Glu83 and Glu87), the S_45_A (Lys294), and the HB (Lys467) helices of K_Ca_2.2 are present (Fig. 4a).

Large differences are apparent in the alignment of CaM’s a-carbons (rmsd = 2.6 Å, Fig. 4b,c) in rimtuzalcap_K_Ca_2.2_I versus apo_K_Ca_2.2. Binding of rimtuzalcap expands CaM’s N-lobes, shortens the distances between the N-lobes in opposite subunits from ~35 Å in apo_K_Ca_2.2 (Fig. 1c) to ~22 Å in rimtuzalcap_K_Ca_2.2_I, and stabilizes K_Ca_2.2’s cytoplasmic HC helices, rendering them visible (Fig. 4d). These observations suggest that a substantial conformational change is required to accommodate rimtuzalcap into its binding pocket in K_Ca_2.2/CaM. In contrast, the conformational change required to fit NS309 into its binding pocket in K_Ca_2.2 channels is minimal because comparison of the NS309_K_Ca_2.2 versus apo_K_Ca_2.2 structures shows CaM’s a-carbons in good alignment (rmsd = 1.4 Å, Supplementary Fig. 7a,b), small changes in the distances between opposite CaM’ N-lobes (~31 Å versus ~35 Å, Figs. 1c, 2c), and cytoplasmic HC helices that remain flexible and invisible (Figs. 1c, 2c).

**Figure 5:**
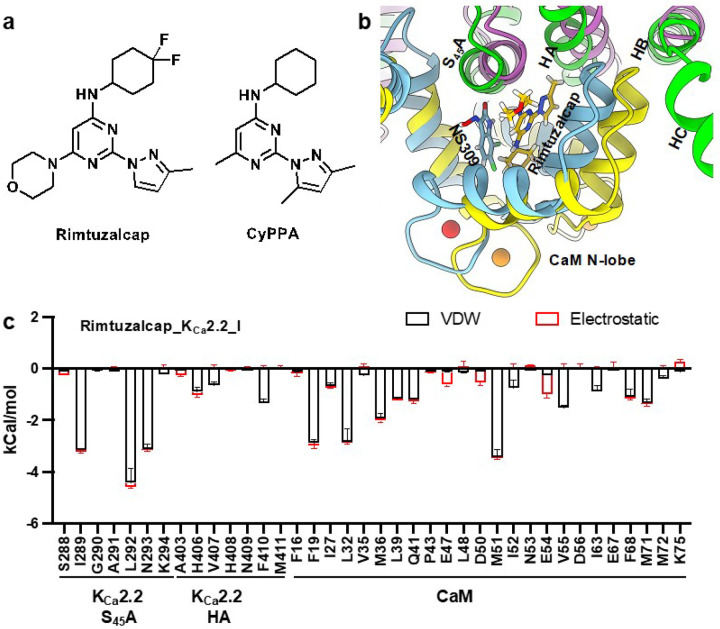
Calculated binding energy between rimtuzalcap and the binding pocket in Rimtuzalcap_K_Ca_2.2_I. a Chemical structures of rimtuzalcap and CyPPA. b The binding pocket of rimtuzalcap in rimtuzalcap_K_Ca_2.2_I (K_Ca_2.2: green/CaM: yellow) superimposed onto the binding pocket of NS309 in NS309_K_Ca_2.2 (K_Ca_2.2: purple/CaM: light blue). Rimtuzalcap forms contacts with both the S_45_A and HA helices, while NS309 primarily interacts with the S_45_A helix. c Binding energy between rimtuzalcap and amino acid residues in CaM and the S_45_A helix of K_Ca_2.2. The total binding energy between rimtuzalcap and the four subunits of the activator-bound structure include van der Waals forces (VDW, black) and electrostatic interactions (Electrostatic, red).

NS309 primarily interacts with K_Ca_2.2’s S_45_A helix (Fig. 3), whereas the bulkier rimtuzalcap (Fig. 5a) forms contacts with K_Ca_2.2’s HA helix in addition to the S_45_A helix (Fig. 5b). Rimtuzalcap fits perfectly into the hydrophobic pocket formed by residues in K_Ca_2.2’s S_45_A helix (Ile289, Leu292, Asn293), K_Ca_2.2’s HA helix in a neighboring subunit (His406, Phe410), and CaM’s N-lobe (Phe19, Leu32, Met36, Met51) (Fig. 5c). The binding energy between rimtuzalcap and its induced-fit binding pocket in K_Ca_2.2 (−36.9 ± 1.3 kCal/mol) is stronger than NS309’s binding energy to its pre-existing binding pocket in K_Ca_2.2 (−27.6 ± 1.0 kCal/mol). However, NS309 is a more potent activator of K_Ca_2.2 (EC_50_: ~1.7 mM) than rimtuzalcap (EC_50_: ~5.1 mM) in electrophysiological assays. The discordance between binding energies and activation-potency of these two compounds may be due inherent variations in ligand-binding to preexisting (NS309) versus induced-fit (rimtuzalcap) pockets^20^ and to differences in ligand efficiency caused by differences in size of the two molecules (Figs. 3d, 5a).

**Figure 6:**
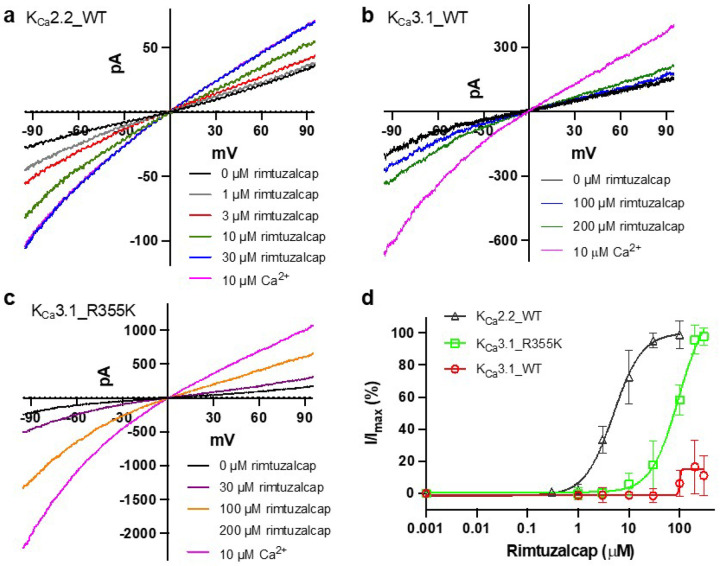
The sensitivity of K_Ca_2.2 and K_Ca_3.1 channels to rimtuzalcap. a Representative current traces of concentration-dependent activation of the K_Ca_2.2_WT channel by rimtuzalcap in inside-out patch clamp recordings in the presence of 0.15 mM Ca^2+^. b Limited responses to rimtuzalcap of the K_Ca_3.1_WT channel in inside-out patch clamp recordings in the presence of 0.15 mM Ca^2+^. c Representative current traces of concentration-dependent activation of the K_Ca_3.1_R355K mutant channel by rimtuzalcap in inside-out patch clamp recordings in the presence of 0.15 mM Ca^2+^. d Responses of K_Ca_3.1_WT and K_Ca_3.1_R355K channels versus K_Ca_2.2_WT channels to rimtuzalcap. Rimtuzalcap activates K_Ca_3.1_R355K (EC_50_: 76.8 ± 21.3 mM, n=8) with ~15-fold lower potency than K_Ca_2.2_WT (EC_50_: 5.1 ± 1.0 mM, n=5, P<0.0001, unpaired two-tailed Student’s *t*-test). The responses were normalized by the maximal currents induced by 10 μM Ca^2+^.

Unlike the K_Ca_2.2 channel (Fig. 6a), the K_Ca_3.1 channel is insensitive to rimtuzalcap (Fig. 6b). In the apo_K_Ca_3.1_II structure, CaM’s N-lobes are close to each other (~27.0 Å apart, Fig. 1d) and the putative binding pocket in CaM’s N-lobe is constrained by the HC helices, which may prevent it from undergoing conformational changes required to accommodate rimtuzalcap. We therefore wondered if rearranging the salt bridges between K_Ca_3.1’s HB helix and CaM’s helix V would release the putative binding pocket from the constraints of the HC helices and allow rimtuzalcap binding. To test this idea, we mutated Arg355 in K_Ca_3.1’s HB helix to lysine to mimic the salt bridge-forming Lys467 in K_Ca_2.2 (Fig. 4a). In support of our idea, the mutant K_Ca_3.1_R355K channel was activated by rimtuzalcap with an EC_50_ = 76.8 ± 21.3 mM (n=8; Fig. 6c,d). Additional structural determinants may underlie rimtuzalcap’s ~15-fold lower potency on K_Ca_3.1_R355K compared to K_Ca_2.2_WT (EC_50_: 5.1 ± 1.0 mM, n=5, P<0.0001, unpaired two-tailed Student’s *t*-test).

### Widening of the inner gate by NS309 and rimtuzalcap

**Figure 7:**
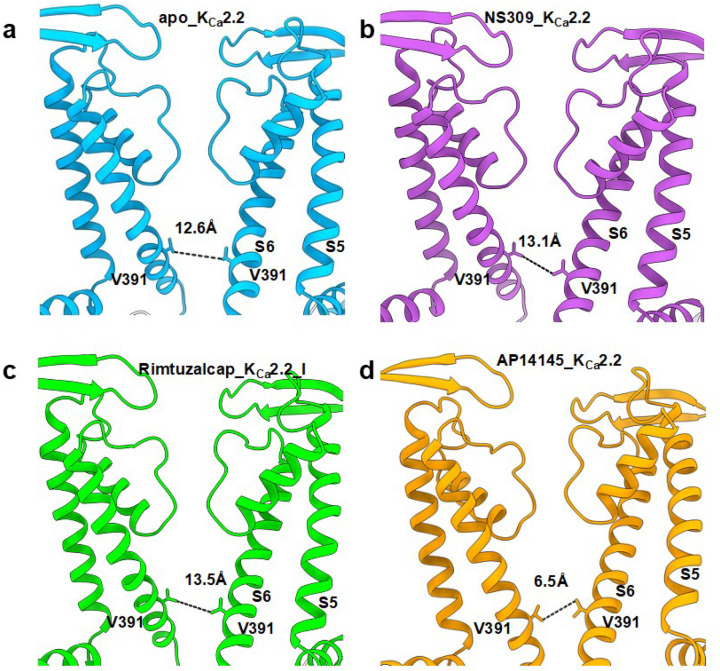
Inner gate of K_Ca_2.2 channels regulated by pharmacological agents. In side views, dimensions of the inner gate are measured as distances between Val391 in the transmembrane S6 helices of opposite K_Ca_2.2 subunits in a apo_K_Ca_2.2, b NS309_K_Ca_2.2, c rimtuzalcap_K_Ca_2.2_I, and d AP14145_K_Ca_2.2 structures. Two opposite channel subunits are shown for clarity.

Rimtuzalcap and NS309 widened K_Ca_2.2’s inner gate from ~12.6 Å in the apo-form (apo_K_Ca_2.2) to ~13.5 Å and ~13.1 Å in the rimtuzalcap_K_Ca_2.2_I and NS309_K_Ca_2.2 structures, respectively (Fig. 7). In our previous cryo-EM study, the inhibitor AP14145 narrowed the inner gate to ~6.5 Å (PDB: 8v2h; referred to henceforth as AP14145_K_Ca_2.2; Fig. 7). Thus, rimtuzalcap and NS309 both activate K_Ca_2.2 by widening the inner gate. Differences in their interactions with the Ca^2+^-CaM-dependent gating machinery of K_Ca_2.2 versus K_Ca_3.1 likely underlie rimtuzalcap’s K_Ca_2.x-selectivity and NS309’s non-selective activation of both K_Ca_2.2 and K_Ca_3.1.

Activators of K_Ca_2.2 have been termed positive allosteric modulators because they work only in the presence of Ca^2+^.^19,21,22^ Both NS309 and rimtuzalcap (Fig. 6) require a minimal concentration (~0.1–0.2 mM) of Ca^2+^ to enhance the activity of K_Ca_2.2 channels. This requirement for Ca^2+^ is illustrated by comparison of two rimtuzalcap-bound K_Ca_2.2 structures built using cryo-EM maps of two 3D classes from the same dataset (Supplementary Fig. 8, 9). The density for rimtuzalcap is visible in both rimtuzalcap_K_Ca_2.2_I and rimtuzalcap_K_Ca_2.2_II (Supplementary Fig. 10), whereas Ca^2+^ ions are present only in CaM’s N-lobes of rimtuzalcap_K_Ca_2.2_I (Supplementary Fig. 10a,b) and not rimtuzalcap_K_Ca_2.2_II (Supplementary Fig. 10c-e). Rimtuzalcap_K_Ca_2.2_II that lacks Ca^2+^ in CaM’s N-lobes exhibits a closed inner gate (~6.9 Å), whereas rimtuzalcap_K_Ca_2.2_I that contains Ca^2+^ in CaM’s N-lobes (Supplementary Fig. 10f,g) exhibits a widened inner gate (~13.5 Å). Taken together, these results suggest that binding of NS309 and rimtuzalcap to the Ca^2+^-bound CaM N-lobe stabilizes its interaction with K_Ca_2.2’s S_45_A helix and dilates the channel’s inner gate.

## Discussion

Even though K_Ca_2.2 and K_Ca_3.1 channels share a Ca^2+^/CaM-dependent gating mechanism, CaM interacts differently with the cytoplasmic HC helices of K_Ca_2.2 and K_Ca_3.1. In apo_K_Ca_2.2, the CaM N-lobes in opposite subunits are too far apart to stabilize K_Ca_2.2’s HC helices (Fig. 1c). In apo_K_Ca_3.1_II, in contrast, the CaM N-lobes in opposite subunits are close enough to stabilize K_Ca_3.1’s HC helices (Fig. 1d). The distinct conformations of CaM in the two channel structures underlie the subtype-selectivity of pharmacological activators.

Accommodation of NS309 into its binding pockets in K_Ca_2.2 and K_Ca_3.1 does not require a prominent conformational change within CaM as evidenced by the excellent alignment of CaM’s a-carbons between the apo- and NS309-bound K_Ca_2.2 and K_Ca_3.1 structures (Supplementary Fig. 7). This indicates that NS309 fits into pre-existing binding pockets in both NS309_K_Ca_3.1 and NS309_K_Ca_2.2. NS309’s stronger electrostatic interactions with CaM_Lys75 (Figs. 3a,b) accounts for its higher total binding energy to NS309_K_Ca_3.1 versus NS309_K_Ca_2.2 (Fig. 3c). This difference in binding strength likely underlies NS309’s ~20-fold higher potency in activating K_Ca_3.1 than K_Ca_2.2 channels.^16,17^

In contrast, accommodation of rimtuzalcap into its binding pockets in K_Ca_2.2 requires significant conformational changes within CaM as evidenced by large differences in the alignment of CaM’s a-carbons between the apo_K_Ca_2.2 and rimtuzalcap_K_Ca_2.2_I structures (Figs. 4b,c). These results suggest that the induced-fit binding pocket of rimtuzalcap does not exist in apo_K_Ca_2.2. Binding of rimtuzalcap expands CaM’s N-lobes around rimtuzalcap and shortens the distances between opposite CaM’s N-lobes, resulting in rigidification of K_Ca_2.2’s cytoplasmic HC helices (Fig. 4d). These activator-induced changes in rimtuzalcap_K_Ca_2.2_I are reminiscent of the mechanism of action of AUT5, an activator of the voltage-gated K_V_3.1 channel. Binding of AUT5 to K_V_3.1 rearranges the turret region around the activator molecule, thereby inducing interactions between the turret and the transmembrane S4 segment in the voltage-sensor domain.^4^

Previously, we reported that CyPPA, an analog of rimtuzalcap, exhibited subtype-selectivity for K_Ca_2.2 over K_Ca_3.1 due to the difference of a single residue in the HB helix (K_Ca_2.2_K467 versus K_Ca_3.1_R355).^23^ However, our earlier docking studies were based on a homology model of K_Ca_2.2 generated with K_Ca_3.1 cryo-EM structures as templates.^12^ Docking into this homology model placed CyPPA in a pre-existing binding pocket between CaM’s C-lobe and K_Ca_2.2’s HA/HB helices^23^. This placement is most likely wrong because CyPPA and rimtuzalcap are structurally very similar (Fig. 5a), and we have shown above that rimtuzalcap fits into an induced-fit binding pocket that emerges as a result of conformational changes in CaM (Fig. 4).

The K_Ca_3.1 channel is insensitive to rimtuzalcap. Our structural, mutagenesis and electrophysiological studies suggest that this insensitivity to rimtuzalcap is because the putative activator binding pocket in CaM’s N-lobe of K_Ca_3.1 is constrained by the HC helices and is prevented from undergoing the requisite conformational changes to accommodate rimtuzalcap. Replacing K_Ca_3.1_R355 with lysine (corresponding to K_Ca_2.2_K467) in K_Ca_3.1’s HB helix frees the binding pocket from the constraints of the HC helices, and allows rimtuzalcap to activate the mutant K_Ca_3.1_R355K channel (EC_50_: ~76.8 mM, Fig. 6).

In conclusion, our structures provide a foundation for understanding the subtype-selectivity of the K_Ca_2.2 activator rimtuzalcap and could enable structure-based drug design of more potent, subtype-selective activators targeting K_Ca_2.2 channels. Subtype-selective K_Ca_2.2 activators that avoid potential side effects associated with activation of peripheral K_Ca_3.1 channels are critically needed to target K_Ca_2.2 channels in the central nervous system for the treatment of spinocerebellar ataxia and essential tremor.

## Methods

### Protein expression and purification

The rat K_Ca_2.2/CaM protein complex was expressed and purified as described in our previous report.^11^ Briefly, the cDNA of full-length rat K_Ca_2.2 (accession no. NM_019314) with a C-terminus Strep-II tag was sub-cloned into pEG BacMam (a gift from Eric Gouaux; Addgene plasmid # 160451; http://n2t.net/addgene:160451; RRID:Addgene_160451). Un-tagged rat calmodulin (CaM) cDNA (accession no. BC063187) was also cloned into pEG BacMam. The amino acid sequence of the *rat* CaM is 100% identical to the *human* CaM. We expressed the K_Ca_2.2/CaM protein complex in HEK293S GnTI− cells (ATCC) using a BacMam method^24^. The K_Ca_2.2/CaM complex was purified using Strep-Tactin XT resin, followed by size exclusion chromatography column equilibrated by 20 mM Tris pH 8, 150 mM KCl, 2 mM CaCl_2_, and 0.01% lauryl maltose neopentyl glycol (LMNG, Anatrace). The peak fractions were collected and concentrated to ~3 mg/ml.

The human K_Ca_3.1/CaM protein complex was expressed and purified as described in previous reports.^12,25^ Briefly, the cDNA of full-length human K_Ca_3.1 (accession no. NM_002250.3) with a C-terminus Strep-II tag was sub-cloned into pEG BacMam. We expressed the K_Ca_3.1/CaM protein complex in HEK293S GnTI− cells (ATCC) using a BacMam method^24^. The K_Ca_3.1/CaM complex was purified using Strep-Tactin XT resin, followed by size exclusion chromatography column equilibrated by 20 mM Tris pH 8, 150 mM KCl, 2 mM CaCl_2_, and 0.007% glyco-diosgenin (GDN, Anatrace). The peak fractions were collected and concentrated to ~3 mg/ml.

### Cryo-EM Sample Preparation, Data Collection and Processing

We performed cryo-EM data collection at the Stanford SLAC Cryo-EM Center (S^2^C^2^) and Pacific Northwest Cryo-EM Center (PNCC). To determine the NS309-bound structures, saturating concentrations of NS309 were mixed with the K_Ca_3.1/CaM or K_Ca_2.2/CaM protein complexes 30 minutes before the grid preparation, respectively. To determine the rimtuzalcap-bound structure, saturating concentrations of rimtuzalcap were mixed with the K_Ca_2.2/CaM protein complex 30 minutes before the grid preparation. Because K_Ca_3.1 channels are not sensitive to rimtuzalcap, the K_Ca_3.1/CaM protein complex was not mixed with rimtuzalcap.

3 μl of purified protein was applied to a glow-discharged Quantifoil R1.2/1.3 300 mesh Copper grid, at 4 °C and 100% humidity using Vitrobot Mark IV (ThermoFisher Scientific). The grid was then blotted for 3 seconds before being plunged into liquid ethane. Grids of NS309-bound K_Ca_3.1/CaM were screened, and cryo-EM data were subsequently collected on a Titan Krios G3i (ThermoFisher Scientific) with a K3 detector (Gatan) and a BioQuantum energy filter. Dose fractionated movies were collected using EPU at the pixel size of 0.86 Å. A total cumulative dose of ~50 electrons per Å^2^ was used for recording the movies of 40 frames (1.25 electrons per Å^2^ per frame) with a defocus range of −1.0 to −2.0 μm.

Grids of rimtuzalcap-bound K_Ca_2.2/CaM were screened, and cryo-EM data were subsequently collected on a Titan Krios G3i (ThermoFisher Scientific) with a K3 detector (Gatan) and a BioQuantum energy filter. Dose fractionated movies were collected using EPU at the pixel size of 0.86 Å. A total cumulative dose of ~50 electrons per Å^2^ was used for recording the movies of 40 frames (1.25 electrons per Å^2^ per frame) with a defocus range of −1.3 to −2.3 μm.

Grids of NS309-bound K_Ca_2.2/CaM were screened, and cryo-EM data were subsequently collected on a Titan Krios G3i (ThermoFisher Scientific) with a Falcon4i detector (ThermoFisher Scientific) and a SelectrisX energy filter. Dose fractionated movies were collected using EPU at the pixel size of 0.73 Å. A total cumulative dose of ~50 electrons per Å^2^ was used for recording in the EER format with a defocus range of −0.6 to −2.2 μm. The statistics for data collections are summarized in Supplementary Tables 1.

Cryo-EM image processing for NS309-bound K_Ca_2.2/CaM was carried out using CryoSparc version 4.5.3^26^. After preprocessing of micrographs (motion correction and CTF estimation), ~2,000 particles were manually picked, followed by 2D classification to generate picking templates. Template-picked particles were cleaned up by multiple rounds of 2D classification. An *ab initio* reconstruction was performed, followed by heterogenous and non-uniform refinements to 2.72 Å resolution in the CryoSparc^26^ program. The cryo-EM map with 2.72 Å resolution was improved by 3D classification followed by nonuniform refinement to 2.71 Å resolution (NS309_K_Ca_2.2, Supplementary Fig. 1). The cryo-EM density for NS309 is clearly visible in the refined map of NS309_K_Ca_2.2 (Fig. 2a and Supplementary Fig. 5).

The NS309-bound K_Ca_3.1/CaM dataset was similarly processed using the CryoSparc program^26^. An *ab initio* reconstruction was performed, followed by heterogenous and non-uniform refinements to 3.69 Å resolution in the CryoSparc^26^ program. The cryo-EM map with 3.69 Å resolution was improved by 3D classification followed by nonuniform refinement to 3.59 Å resolution (NS309_K_Ca_3.1, Supplementary Fig. 3). The cryo-EM density for NS309 is clearly visible in the refined map of NS309_K_Ca_3.1 (Fig. 2b and Supplementary Fig. 6).

For the rimtuzalcap-bound K_Ca_2.2/CaM dataset, an *ab initio* reconstruction was performed, followed by heterogenous and non-uniform refinements to 2.95 Å resolution in the CryoSparc^26^ program. To examine possible conformational heterogeneity, 3D classification was performed. Two classes were generated by 3D classification, which were further refined using non-uniform refinements to 3.13 Å resolution (rimtuzalcap_K_Ca_2.2_I), and 2.96 Å resolution (rimtuzalcap_K_Ca_2.2_II), which were used for model building (Supplementary Fig. 8).

The cryo-EM density of rimtuzalcap is clearly visible at the interface between the CaM N-lobe and the S_45_A/HA helices of K_Ca_2.2 in both rimtuzalcap_K_Ca_2.2_I and rimtuzalcap_K_Ca_2.2_II (Supplementary Fig. 10). The cryo-EM densities of two Ca^2+^ ions are visible at the CaM N-lobes of rimtuzalcap_K_Ca_2.2_I but not in rimtuzalcap_K_Ca_2.2_II. In a portion of rimtuzalcap-bound K_Ca_2.2 channels, the CaM N-lobe may lose Ca^2+^ binding (rimtuzalcap_K_Ca_2.2_II), which in turn closes the inner gate because Ca^2+^ binding to the CaM N-lobe is required for the activation of K_Ca_2.2^22^. The other portion of rimtuzalcap-bound K_Ca_2.2 channels may retain both Ca^2+^ and rimtuzalcap (rimtuzalcap_K_Ca_2.2_I), which exhibits a wider inner gate (~13.5 Å) than the apo_K_Ca_2.2 structure (~12.6 Å). Since Ca^2+^ binding to the CaM N-lobe is required for the activation of K_Ca_2.2^22^, rimtuzalcap_K_Ca_2.2_I that contains both rimtuzalcap and Ca^2+^ was used for further analysis of the interactions between rimtuzalcap and K_Ca_2.2 channels.

Resolutions were estimated using the gold standard criterion at the threshold of 0.143. The local resolution was calculated in CryoSparc.

### Model building

Coordinates of the Ca^2+^-bound K_Ca_2.2 (PDB: 8v2g; apo_K_Ca_2.2) were used as the initial model for activator-bound K_Ca_2.2 structures. Coordinates of the Ca^2+^-bound K_Ca_3.1 activated state II (PDB: 6cno; apo_K_Ca_3.1_II) were used as the initial model for activator-bound K_Ca_3.1 structures. The initial model was manually docked into the cryo-EM density map and then adjusted in UCSF Chimera.

Phenix.real_space_refine^27^ was used to build and refine the model. Model building was achieved using phenix^27^ and Coot^28^ iteratively. Models for Ca^2+^, K^+^, and activators were built by visual inspection of the shape of the density in Coot^28^ followed by refinement in phenix^27^. The statistics for model refinements are summarized in Supplementary Table 1. All structural graphics were generated using UCSF ChimeraX^29^.

### Patch-clamp electrophysiology

Human Embryonic Kidney (HEK293) cells transiently transfected with the rat K_Ca_2.2 or human K_Ca_3.1 cDNAs were used for manual patch-clamp experiments. Site-directed mutagenesis of the K_Ca_3.1_R355K was performed on the cDNAs through molecular cloning services (Genscript). The wildtype and mutant cDNAs, constructed in the pIRES2-AcGFP1 vector (Clontech),were transfected into HEK293 cells by a calcium–phosphate method. Inside-out K_Ca_2.2 currents were recorded 1–2 days after transfection, with an Axon200B amplifier (Molecular Devices) at room temperature.

The resistance of the patch electrodes ranged from 2–3 MΩ. For inside-out recordings, the intracellular solution containing (in mM): 140 KCl, 10 Hepes (pH 7.2), 1 EGTA, 0.1 Dibromo-BAPTA, and 1 HEDTA was mixed with Ca^2+^ to obtain the desired free Ca^2+^ concentrations, calculated using the MaxChelator software. The extracellular solution contained (in mM): 140 KCl, 10 Hepes (pH 7.4), 1 MgSO_4_.

NS309 (6,7-dichloro-1*H*-indole-2,3-dione 3-oxime) was purchased from Alomone labs. NS309 dilutions were prepared freshly in extracellular solution from 20 mM stock solutions in DMSO.

Rimtuzalcap (also called CAD-1883, *N*-(4,4-difluorocyclohexyl)-2-(3-methylpyrazol-1-yl)-6-morpholin-4-ylpyrimidin-4-amine) was purchased from MedChemExpress. Rimtuzalcap dilutions were prepared freshly in extracellular solution from 100 mM stock solutions in DMSO.

pClamp 10.5 (Molecular Devices) was used for data acquisition and analysis. To characterize the responses of the K_Ca_3.1_R355K mutants to rimtuzalcap, inside-out patch recordings were performed. Seals (> 1 GΩ) were formed before the inside-out patch configuration was obtained. The intracellular face was exposed to a series of activator concentrations at a fixed Ca^2+^ concentration (0.15 mM Ca^2+^). Currents were recorded by repetitive 1s voltage ramps from − 100 mV to 100 mV from a holding potential of 0 mV. One minute after switching of bath solutions, ten sweeps with a 1 s interval were recorded. To construct the concentration-dependent activation of channel activities, the current amplitudes at − 90 mV in response to various concentrations of activatorswere normalized to that obtained at 10 mM of Ca^2+^. The normalized currents were plotted as a function of the concentrations of the activators. EC_50_ values and Hill coefficients were determined by fitting the data points to a standard concentration–response curve.

Data analysis was performed using pClamp 10.5 (Molecular Devices) in a blinded fashion. Concentration-response curves were analyzed in GraphPad Prism 10 (GraphPad Software Inc.). All data are shown as mean ± SD unless otherwise indicated. One-way ANOVA and Tukey’s post hoc tests were used for data comparison of three or more groups. The unpaired two-tailed Student’s *t*-test was used for data comparison if there were only two groups. Figures were made using GraphPad Prism 10 (GraphPad Software Inc.).

### Calculation of binding energy

The binding energy between NS309 and rimtuzalcap in their binding pockets were calculated using the cryo-EM structures and the Discovery Studio program (Dassault Systemes Biovia LLC). Briefly, the channel complex structures were subjected to energy minimization using Smart Minimizer algorithm (200 steps) and Generalized Born (GB) Implicit Solvent and Membrane model using the CHARMm forcefield. Interaction energies between the drugs and channels were calculated using an Implicit Distance-Dependent Dielectrics (Dielectric Constant=2.0) solvent model. Residues within 6.5 Å from the drugs were selected for interaction energy and energy decomposition calculations.

## Supplementary Files

This is a list of supplementary files associated with this preprint. Click to download.

- Validationreports.pdf
- RimSupplementary.pdf

## Data Availability

The atomic coordinates have been deposited in the Protein Data Bank (PDB) under accession codes 9O7S [https://doi.org/10.2210/pdb9O7S/pdb] (NS309_K_Ca_2.2); 9OA8 [https://doi.org/10.2210/pdb9OA8/pdb] (NS309_K_Ca_3.1); 9O85 [https://doi.org/10.2210/pdb9O85/pdb] (rimtuzalcap_K_Ca_2.2_I); and 9O93 [https://doi.org/10.2210/pdb9O93/pdb] (rimtuzalcap_K_Ca_2.2_II). The cryo-EM maps have been deposited in the Electron Microscopy Data Bank (EMDB) under accession codes EMD-70207 [https://www.ebi.ac.uk/pdbe/entry/emdb/EMD-70207] (NS309_K_Ca_2.2); EMDB-70275 [https://www.ebi.ac.uk/pdbe/entry/emdb/EMDB-70275] (NS309_K_Ca_3.1); EMD-70217 [https://www.ebi.ac.uk/pdbe/entry/emdb/EMD-70217] (rimtuzalcap_K_Ca_2.2_I); and EMD-70240 [https://www.ebi.ac.uk/pdbe/entry/emdb/EMD-70240] (rimtuzalcap_K_Ca_2.2_II). The following previously published PDB codes are referred to: 8v2g [https://doi.org/10.2210/pdb8v2g/pdb]; 8v2h [https://doi.org/10.2210/pdb8v2h/pdb]; 6cnn [https://doi.org/10.2210/pdb6cnn/pdb]; 6cno [https://doi.org/10.2210/pdb6cno/pdb]. All other data supporting the findings of this study are available within the paper and its Supplementary information files.
